# Influences of Canopy Nitrogen and Water Addition on AM Fungal Biodiversity and Community Composition in a Mixed Deciduous Forest of China

**DOI:** 10.3389/fpls.2018.01842

**Published:** 2018-12-11

**Authors:** Aihua Zhao, Lei Liu, Tianle Xu, Leilei Shi, Wei Xie, Wei Zhang, Shenglei Fu, Haiyan Feng, Baodong Chen

**Affiliations:** ^1^State Key Laboratory of Urban and Regional Ecology, Research Center for Eco-Environmental Sciences, Chinese Academy of Sciences, Beijing, China; ^2^University of Chinese Academy of Sciences, Beijing, China; ^3^Global Ecology Unit, Facultat de Biociencies, CREAF-CSIS-UAB, Barcelona, Spain; ^4^Laboratory of Geospatial Technology for the Middle and Lower Yellow River Regions, College of Environment and Planning, Henan University, Kaifeng, China; ^5^Key Laboratory of Vegetation Restoration and Management of Degraded Ecosystems, South China Botanical Garden, Chinese Academy of Sciences, Guangzhou, China; ^6^School of Earth Sciences and Resources, China University of Geosciences, Beijing, China

**Keywords:** nitrogen deposition, precipitation, AM fungi, forest ecosystem, community composition, climate change, canopy

## Abstract

Nitrogen (N) deposition and precipitation could profoundly influence the structure and function of forest ecosystems. However, conventional studies with understory additions of nitrogen and water largely ignored canopy-associated ecological processes and may have not accurately reflected the natural situations. Additionally, most studies only made sampling at one time point, overlooked temporal dynamics of ecosystem response to environmental changes. Here we carried out a field trial in a mixed deciduous forest of China with canopy addition of N and water for 4 years to investigate the effects of increased N deposition and precipitation on the diversity and community composition of arbuscular mycorrhizal (AM) fungi, the ubiquitous symbiotic fungi for the majority of terrestrial plants. We found that (1) in the 1st year, N addition, water addition and their interactions all exhibited significant influences on AM fungal community composition; (2) in the 2nd year, only water addition significantly reduced AM fungal alpha-diversity (richness and Shannon index); (3) in the next 2 years, both N addition and water addition showed no significant effect on AM fungal community composition or alpha-diversity, with an exception that water addition significantly changed AM fungal community composition in the 4th year; (4) the increment of N or water tended to decrease the abundance and richness of the dominant genus *Glomus* and favored other AM fungi. (5) soil pH was marginally positively related with AM fungal community composition dissimilarity, soil NH_4_^+^-N and N/P showed significant/marginal positive correlation with AM fungal alpha-diversity. We concluded that the effect of increased N deposition and precipitation on AM fungal community composition was time-dependent, mediated by soil factors, and possibly related to the sensitivity and resilience of forest ecosystem to environmental changes.

## Introduction

Arbuscular mycorrhizal (AM) fungi can form mutualistic symbioses with the majority of terrestrial plants (Smith and Read, 2008) and provide vital ecological services such as improving plant mineral nutrition (Li et al., 2006; Subramanian et al., 2006), enhancing plant tolerance to biotic (Elsen et al., 2008; Affokpon et al., 2011) and abiotic stresses (e.g., flooding, high temperature) (Fougnies et al., 2007; Li et al., 2009; Zhu et al., 2011; Camprubi et al., 2012), altering the composition and diversity of plant communities and influencing the productivity, structure and stability of ecosystems (van Der Heijden et al., 1998, 2008; Jansa et al., 2008), and intensifying the resilience of ecosystem to global climate change (Martínez-García et al., 2017). In view of their ecological significance, investigation on AM fungal diversity and community assemblage has become hot topics in soil ecology in recent years. AM fungal community assembly could be predicted by both niche theory which assumes that the competition among species for limited resources and the differentiation of niche space across species allow species coexistence, emphasizing the importance of determined processes in structuring community assembly (Leibold and McPeek, 2006), and neutral theory which presumes that all species are ecologically equivalent, emphasizing the significance of stochastic processes and dispersal limitation depending on spatial scales (Hubbell, 2001). At global and regional scales, neutral theory weighs more than the ecological niche theory, and AM fungal distribution pattern is mainly shaped by geographical distance and climate factors. However, the ecological niche theory dominates at local scale and small scale, and the effects of host plants and the soil properties on AM fungal community become more important than geographical distance restriction (Chen et al., 2018).

In recent decades, global climate change driven by anthropogenic disturbance has been intensified and impacted the structure and function of multiple aquatic and terrestrial ecosystems (Marino et al., 2017). As an important component of global climate change, nitrogen (N) deposition and its ecological consequences have attracted serious concerns. At global scale, it is estimated that the N deposition rate has increased nearly 34 Tg N yr^-1^ in 1860–100 Tg N yr^-1^ in 1995 and may increase up to 200 Tg N yr^-1^ in 2050 (Galloway et al., 2004). Increased N deposition could cause negative effects on terrestrial ecosystems, such as biodiversity loss, soil acidification, productivity decline, nutrient imbalance and forest degradation (Vitousek et al., 1997; Magill et al., 2004; Hogberg et al., 2006; Bobbink et al., 2010; Lu et al., 2014). Most previous studies on N deposition are carried out in Europe and North America, while studies on N deposition and its ecological consequences in China are rather limited. As a fact, with the rapid development, China is also experiencing increasing N deposition, especially in its central and southeastern areas (Jia et al., 2014). The mean wet N deposition over China has increased nearly 25% from 1990s to 2000s (Jia et al., 2014), and the N deposition rate in China is predicted to continually increase in the coming decades (Liu et al., 2013).

Besides increased N deposition, changes in precipitation patterns is also an important component of global change. According to IPCC report, heavy precipitation events including the frequency and intensity of heavy precipitation over land regions increased markedly in recent years. In many mid-latitude regions, mean precipitation will gradually increase in the 21st century (Integrated Professional Competency Course [IPCC], 2013). Increased precipitation could change species richness and alter the plant community structure and aboveground net primary productivity (ANPP) in arid and semi-arid (water-limited) steppe ecosystems (Yang et al., 2011; Zeppel et al., 2014; Ren et al., 2015). Intensified precipitation can also elevate the risk of soil nutrient leaching (e.g., N, P) (Martínez-García et al., 2017). Moreover, under natural conditions, multiple global changes may occur simultaneously and interact with each other (Harpole et al., 2007). The impacts of N deposition on the ecosystem structure and function would be substantially altered by precipitation (Harpole et al., 2007; Yang et al., 2011; Araya et al., 2013). Previous studies showed that precipitation increment could alleviate the negative effects of increased N deposition by increasing the mobility and leaching of soil inorganic N (Li et al., 2016; Sun et al., 2017). More extensive studies on the interactive effects of N deposition and precipitation on ecosystems are still expected.

Up to date, very limited information is available as for the responses of belowground ecosystem especially for soil microbial communities, to climate changes (Li et al., 2016). Definitely more attention should be paid to the soil microorganisms which play important roles in nutrient cycling, organic matter decomposition, primary production, regulation of greenhouse emissions and other ecosystem functions (Philippot et al., 2013; Wagg et al., 2014; Delgado-Baquerizo et al., 2017). As an important functional group of soil microbes, AM fungi directly bridge up plant and soil, and are selected as model organism for studying belowground-aboveground interactions. It has been well documented that increased N deposition decreased the abundance (van Diepen et al., 2007, 2010; Camenzind et al., 2014, 2016), richness (Camenzind et al., 2014; Liu et al., 2014; Chen et al., 2017) and diversity (Chen et al., 2017), and altered the community composition (van Diepen et al., 2011; Chen et al., 2014; Zheng et al., 2014; Kim et al., 2015) of AM fungi. Precipitation increment can also decrease the abundance and alpha-diversity (Chen et al., 2017), and alter community composition (Gao et al., 2016; Chen et al., 2017) of AM fungi in semiarid (water-limited) steppe ecosystem. However, as far as we know, there were only limited reports on the interaction of N deposition and precipitation on AM fungal communities, and they all were carried out in water-limited steppe ecosystems (Li et al., 2015; Chen et al., 2017). The interaction could be different in humid forest ecosystem from arid/semi-arid steppe ecosystems. In addition, most previous studies on the ecological impacts of precipitation increment or N deposition on forest ecosystems largely ignored many canopy-associated ecological processes by understory addition of N or water. The canopy-associated processes may include N uptake by leaves, epiphytes and microbes; immobilization in decaying leaves or other dead organic matters; volatilization as water evaporates; and transformation of inorganic N to organic N (Zhang et al., 2015). Undoubtedly, canopy processes are more important in forest than in grassland and should not be neglected. As seen in report, the percentage of retained N from N deposition by forest canopy could vary in different studies from 1∼5% to 10∼25%, depending on forest type and N deposition intensity (Zhang et al., 2015).

Furthermore, AM fungal community structure exhibits seasonal dynamics and interannual variability (Husband et al., 2002; Hazard et al., 2014). Husband et al. (2002) investigated the diversity and distribution of AM fungi colonizing tree seedling roots for 2 years in the tropical forest on Barro Colorado Island, Republic of Panama. They found that dominant AM fungal types in the first year were nearly entirely replaced by previously rare types in the following year; Hazard et al. (2014) investigated the effects of biosolids on AM fungal communities in grassland and arable agroecosystems and found that the effect of seasonality exceeded that of biosolids application. The AM fungal community compositions (using T-RFLP method) associated with *Lolium perenne* shifted with seasonality and year, some dominant AM fungi (e.g., T-RFs associated with *Rhizophagus irregularis*) were present in roots throughout and between years, others were only present seasonally (e.g., *HinfI*-HEX 422), and rarer species fluctuated in presence and frequency. However, many studies only made one sampling and overlooked the temporal dynamics of AM fungal community in response to environmental changes.

As a result, we conducted a field trial in a mixed forest in China’s climate transition zone from subtropical to warm temperate climate to investigate the impacts of N deposition and precipitation increment on AM fungal community with canopy N and water addition. We carried out field investigation and collected soil samples every year since the experiment establishment in 2013, and analyzed AM fungal diversity and community structure by using the high throughput sequencing technology. We hypothesized that (1) canopy N or water addition would significantly decrease AM fungal richness, Shannon diversity index and change community composition; (2) N and water addition interactively shape AM fungal community; (3) the effects of N and water addition on AM fungal community would be time-dependent. To the best of our knowledge, this study for the first time investigated the interactive effects of increased N deposition and precipitation on AM fungal diversity and community composition in a forest ecosystem and is expected to allow better understanding of the impacts of climate changes on the forest ecosystems.

## Materials and Methods

### Study Site

The study site was located in Jigongshan (JGS) National Nature Reserve (31°46′-31°52′ N, 114°01′-114°06′ E) representing a transitional zone from subtropical to warm temperate climate in China (Zhang et al., 2015). Dominant tree species in the forest are *Quercus acutissima* Carruth., *Quercus variabilis* Bl. and *Liquidambar formosana* Hance. Both oaks form ectomycorrhiza, while *Liquidambar formosana* Hance can form arbuscular mycorrhiza. In addition, there are some understory shrubs, such as *Lindera glauca* (Sieb.et Zucc.) Bl and *Rubus lambertianus* Ser., and herbs such as *Ophiopogon japonicas* (Linn. f.) Ker-Gawl. and *Phaenosperma globosa* Munro ex Benth. which also can form arbuscular mycorrhiza. The soil type is Ferri-Udic Argosols (yellow brown soils) and the pH value is 5.0-6.0.

According to the meteorological data from 1951 to 2011, the local mean annual temperature (MAT) is 15.2°C and the mean annual precipitation (MAP) is 1119 mm. 80% of the precipitation occurs in April- October. The average annual air humidity is 79%. The background N deposition rate in rainfall is 19.6 kg N ha^-1^yr^-1^, in which the NH_4_^+^/NO_3_^-^ ratio is close to 1 (Zhang et al., 2015; Zhang et al., 2018). More information of JGS Reserve were described by Zhang et al. (2015).

### Experimental Design

This experiment was set up as a fully randomized two-factor block design with four blocks, each block included four plots, and each plot was 17 m in radius. Within each block, each plot was randomly assigned with one of the four treatments: control (CK, ambient environment), canopy addition of N (CN), canopy addition of water (CW), and canopy additions of both N and water (CNW). In order to prevent the interference among treatments, a 20 m buffer zone was left between any two plots and a PVC isolation board with depth of 1 m was installed in the middle of the buffer zone.

The N application rate was 50 kg ha^-1^ yr^-1^, which is predicted to occur in the near future in this region by Liu et al. (2013). The amount of water addition was 30% of the MAP (336 mm), and the magnitude was within the range of model predictions for future precipitation increment induced by warming in Northern Hemisphere by Yang et al. (2015). Nitrogen was added as NH_4_NO_3_ solution (7.7 mmol/L), both the solvent and water are from the local lake. Nitrogen and water were added during the growing season from April to October, and N was applied once a month (totally seven times per year), while water was added once a week (12 mm per week) to prevent surface runoff (Shi et al., 2018). The treatment dates were determined according to the phenology of the forest, i.e., the first time was conducted 1 week before all buds began to open (mid-April), and the last was conducted as leaf litter began to fall (mid-October) (Shi et al., 2016).

All the treatments except CK were realized with a forest canopy spraying system built in the center of the plots. This system can pump N solution or water to a height of 35 m (almost 5 m above the forest canopy) through PVC pipes (10 cm in diameter) which were fixed on the supporting tower. The N solution or water was evenly sprayed onto the forest canopy of the plot by four sprinklers with different spraying range and could be rotated 360° freely (Shi et al., 2018). The working rules and efficiency of the system were described in details by Zhang et al. (2015) and Shi et al. (2016, 2018).

### Soil Sampling and Laboratory Analysis

We made sampling after the seventh treatment every year (mid or late November or December depending on the weather condition). To minimize the impact of sampling position as far as possible, 5 dominant trees which are evenly distributed in the 20 × 20 m^2^ core area of each plot were selected as target trees. Two soil cores (3 cm diameter, 10 cm depth) within the range of 2 m from each target tree, 10 soil cores in total per plot were taken and mixed into one soil sample. The fresh soil samples were transported to laboratory on ice bag, passed through a 2-mm sieve, and divided into 2 subsamples. One was freeze-dried for the DNA extraction, the other was air dried for analysis of soil physicochemical properties.

Soil moisture content was measured gravimetrically by oven-drying the fresh soil samples to constant weight at 105°C. Soil NH_4_^+^-N and NO_3_^-^-N were extracted with 2 mol L^-1^ KCl (a soil to water ratio of 1: 5) and measured using a continuous flow analyzer (SAN++, Skakar, Breda, Holland). Soil available N (AN) was the sum of NH_4_^+^-N and NO_3_^-^-N. Soil pH was determined in a soil/water suspension [1: 2.5 (w/v)] by PB-10 pH-meter (Sartorius, Göttingen, Germany). Soil organic carbon (SOC) was measured according to Walkley and Black (1934). Total N (TN) was determined on an element analyzer (Vario EL Ш[scale=0.5]img001, Elementar, Germany). Soil C/N ratio (C/N) was calculated based on SOC and TN. Soil available phosphorus (AP) was extracted with 0.5 M NaHCO_3_ and measured using a colorimetric method (Murphy and Riley, 1962). Soil N/P ratio (N/P) was calculated based on AN and AP.

Soil DNA was extracted from 0.25 g freeze-dried soil sample by using the Power-Soil^®^ DNA Isolation Kit (MO BIO Laboratories, San Diego, CA, United States) according to the manufacturer’s instructions. Each soil DNA sample was diluted (1:5) with sterilized Milli-Q water. We conducted a nested PCR with primer pairs AML1/AML2 (Lee et al., 2008) and AMV4.5NF/AMDGR (Sato et al., 2005). The first PCR was conducted with a total volume of 25 μl, which contained 2.5 μl 10 × Ex Taq Buffer (Mg^2+^+plus), 2.0 μl dNTP mixture, 0.25 μl Ex Taq (5U/μl)(TaKaRa, Dalian, China), 1.0 μl (10 mg/ml) BSA (TaKaRa, Dalian, China), 0.5 μl (10 μM) of each primer, 17.25 μl sterilized water and 1.0 μl DNA template. The PCR program of amplification are as follows: 94°C for 3 min; 35 cycles at 94°C for 45 s, 51°C for 40 s, 72°C for 1 min; followed by 72°C for 10 min and 16°C for 2 min. The first PCR products were diluted (1:10) with sterilized Milli-Q water, and then used as a template for the second PCR amplification (25 μl) under the following conditions: 94°C for 3 min; 35 cycles at 94°C for 40 s, 58°C for 1 min and 72°C for 1 min, followed by 72°C for 10 min and 4°C for 2 min. The volume of the second PCR was the same as the first. An Eppendorf Mastercycler pro-thermocycler (Eppendorf, Hamburg, Germany) was used for PCR amplification. The PCR products were separated through a 1.5% agarose gel in 1×TAE, bands were excised, and purified with the 0.8×Agencourt AMPure XP Beads (Beckman Coulter Inc., Boulevard Brea, CA, United States). The amount of DNA in the purified PCR products was measured using a Qubit 2.0 Fluorometer (Thermo Fisher Scientific Inc., Hudson, NH, United States). The final products were mixed at equimolar concentrations and then subjected to sequencing on the Illumina MiSeq platform with MiSeq Reagent Kit v3 at Shanghai Hanyu Biotech Co., Ltd.

### Bioinformatics

The initial quality filtering and assembling of paired-end reads were performed by Shanghai Hanyu Biotech Co., Ltd. Raw sequence data were processed in Trimmomatic v 0.32 (Bolger et al., 2014). Main flows are as follows: (1) Remove the reads with N bases; (2) Remove the low-quality bases (*Q* value < 20); (3) Remove the reads itself and its pairing reads whose length is less than 50 bp. Paired-end reads were assembled by Mothur v.1.32.1 (Schloss et al., 2009) permitting 1 bp mismatches of primer bases. Sequences with maxhomop > 8, or shorter than 200 bp were removed. Chimeras were checked in Chimera.uchime v.4.2 (Edgar et al., 2011). OTUs clustering was achieved in Usearch v.9.0.2132_i86linux32 (Edgar, 2010) with a 97% identity threshold. Taxonomic assignment was performed by blasting the representative sequence of each OTU against NCBI GenBank and MaarjAM database (Öpik et al., 2010), a conservative approach was followed for the species identification: considering only identifications with > 97% similarity, >90% coverage and >200 BLAST score value (Supplementary Table S1). In order to further identify the taxonomic information for all AM fungal OTUs, we constructed a neighbor joining phylogenic tree (Supplementary Figure S1) in MEGA v5 (Tamura et al., 2011) with default parameters except that bootstrap replication was set at 1,000 with the Kimura 2 – parameter model. Representative sequences from each encountered AM fungal OTU have been deposited in GenBank (accession numbers MH205770 - MH205915).

### Statistical Analysis

All statistical analyses were conducted in R (R Development Core Team). OTU tables are subsampled to the median according to de Ca’rcer et al. (2011) as the dataset of AM fungal community composition, and then used to calculate the richness and Shannon diversity index (H’). Shannon diversity index (H’) was calculated using the function ‘diversity’ in R package ‘vegan’ (Oksanen et al., 2013). To analyze the effects of canopy N and water addition and their interactions on AM fungal richness, Shannon diversity index (H’) and soil properties, two-way analysis of variance (ANOVA) was conducted, followed by Duncan’s multiple range test. The significant difference was accepted at *P* < 0.05. Before evaluating the effects of canopy N and water additions and their interactions on AM fungal community composition, the data of AM fungal community composition was sqrt transformed, and then a two-way permutational multivariate analysis of variance (PERMANOVA) (Anderson, 2001) was performed using the function ‘adonis2’ in R package ‘vegan’ (Oksanen et al., 2013) with 9999 permutations. In order to further confirm the differences in the composition of AM fungal communities among treatments, we used the functions ‘mrpp’, ‘adonis’ and ‘anosim’ in R package ‘vegan’ (Oksanen et al., 2013) with 9999 permutations. To analyze the influence of soil properties (AP, pH, SOC, TN, C/N, moisture, NH_4_^+^-N, NO_3_^-^-N, AN, N/P) on the AM fungal richness and Shannon diversity index (H’), we performed Pearson correlation analysis. To explore the relationship between AM fungal community composition dissimilarity and soil properties, Mantel and partial Mantel test were carried out using functions ‘mantel’ and ‘mantel.partial’ in R package ‘vegan’ (Oksanen et al., 2013) with 9999 permutations. In all the analyses involved in AM fungal richness, Shannon diversity and soil properties, in order to discover outliers Dixon’s *Q* test was used at 95% confidence level (Dean and Dixon, 1951) and to satisfy the assumption of normality, some soil properties were log or sqrt transformed: in the 1st year, C/N and N/P were log transformed; in the 2nd year, NH_4_^+^-N was sqrt transformed; in the 3rd year, SOC and N/P were log transformed, TN was sqrt transformed; in the 4th year, AP was sqrt transformed, SOC, C/N and AN were log transformed.

## Results

### Effects of Canopy N and Water Addition on Soil Properties

In the 1st year, N addition significantly decreased soil pH. Significant interactive effect of N and water addition on soil C/N was observed: without water addition, N addition had negative effect on soil C/N; with water addition, the effect became positive. In the 2nd year, N addition significantly increased soil NH_4_^+^-N; water addition significantly increased soil AP, but decreased soil NH_4_^+^-N, AN and N/P. To the 3rd and 4th year, there were no significant effect of N or water addition on any soil properties, except that water addition significantly increased AN in the 3rd year (Table 1 and Supplementary Table S2).

**Table 1:** Effects of canopy additions of nitrogen and water on soil properties.

		d.f.		AP (mg/kg)	pH	SOC (g/kg)	TN (g/kg)	C/N	Moisture (%)	NH_4_^+^-N (mg/kg)	NO_3_^-^-N (mg/kg)	AN (mg/kg)	N/P
1st year	CN	1	*F*	0.868	5.597	2.820	0.003	3.333	0.434	0.779	1.118	2.342	1.239
			*p*	0.376	**0.042**	0.132	0.958	0.105	0.527	0.400	0.318	0.160	0.298
	CW	1	*F*	0.151	0.140	0.387	0.649	2.985	0.079	0.559	0.057	0.092	0.928
			*p*	0.707	0.717	0.551	0.441	0.122	0.786	0.474	0.817	0.768	0.364
	CN × CW	1	*F*	0.054	0.579	0.653	1.957	7.602	0.650	0.020	0.898	0.859	0.343
			*p*	0.821	0.466	0.422	0.195	**0.025**	0.441	0.891	0.368	0.378	0.574
2nd year	CN	1	*F*	0.837	1.015	0.107	0.130	0.023	0.542	8.907	0.444	3.574	0.215
			*p*	0.387	0.343	0.751	0.727	0.882	0.480	**0.017**	0.522	0.096	0.657
	CW	1	*F*	5.688	0.232	0.144	0.311	0.735	0.044	22.221	0.113	12.148	7.881
			*p*	**0.044**	0.643	0.714	0.590	0.414	0.839	**0.002**	0.745	**0.008**	**0.026**
	CN × CW	1	*F*	1.227	0.027	0.025	0.299	0.010	0.441	3.903	1.792	3.837	0.024
			*p*	0.300	0.874	0.879	0.598	0.922	0.523	0.084	0.214	0.086	0.882
3rd year	CN	1	F	0.091	0.054	0.984	0.236	1.143	0.473	0.232	0.706	0.603	0.100
			*p*	0.769	0.822	0.347	0.639	0.313	0.509	0.641	0.423	0.457	0.759
	CW	1	*F*	0.387	0.410	3.495	4.076	0.470	0.930	5.089	3.715	7.030	1.961
			*p*	0.549	0.538	0.094	0.074	0.510	0.360	0.051	0.086	**0.026**	0.195
	CN × CW	1	*F*	0.344	0.005	0.094	1.138	0.403	0.682	0.263	0.467	0.360	0.700
			*p*	0.572	0.947	0.766	0.314	0.542	0.430	0.621	0.512	0.563	0.424
4th year	CN	1	*F*	0.000	2.186	0.167	0.115	0.018	1.132	1.626	0.500	1.210	0.946
			*p*	0.983	0.178	0.692	0.743	0.897	0.315	0.234	0.497	0.300	0.359
	CW	1	*F*	2.463	1.540	2.172	2.322	1.267	0.671	1.343	1.431	2.417	2.143
			*p*	0.155	0.250	0.134	0.162	0.290	0.434	0.276	0.262	0.154	0.181
	CN × CW	1	*F*	0.564	0.030	0.542	0.717	0.039	0.009	0.124	0.065	0.017	2.267
			*p*	0.474	0.867	0.480	0.419	0.848	0.928	0.733	0.804	0.898	0.171

*F*-values and *p*-values are presented, significant effects (*p* < 0.05) are highlighted in bold as determined by two-way ANOVA. N, nitrogen addition; W, water addition; N × W, the interaction.

### Overall Miseq-Sequencing Information and OTU Delineation

**FIGURE 1:**
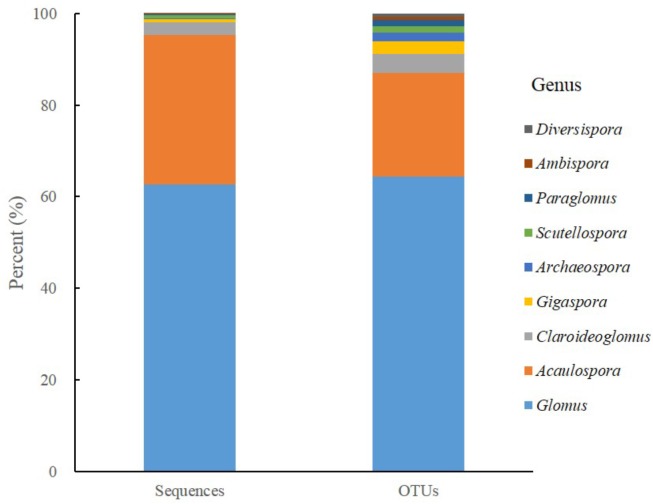
The proportional distributions of sequences and derived AM fungal OTUs detected in all soil samples.

After filtering out chimeras, 624,153 sequences were kept and clustered into 567 OTUs. The sequences of AM fungi accounted for 74.90% (467,479/624,153) of the total sequences and the AM fungal OTUs accounted for 25.75% (146/567) of the total OTUs. Moreover, the majority of AM fungal sequences (62.65%, 292,883/467,479) and OTUs (64.38%, 94/146) belonged to genus *Glomus*, followed by *Acaulospora* sequences (32.58 %, 152,330/467,479) and OTUs (22.60 %, 33/146), while the others covered 7 genera from 6 families, 4 orders (Figure 1).

### Effects of Canopy N and Water Addition on AM Fungal Alpha-Diversity and Community Composition

**FIGURE 2:**
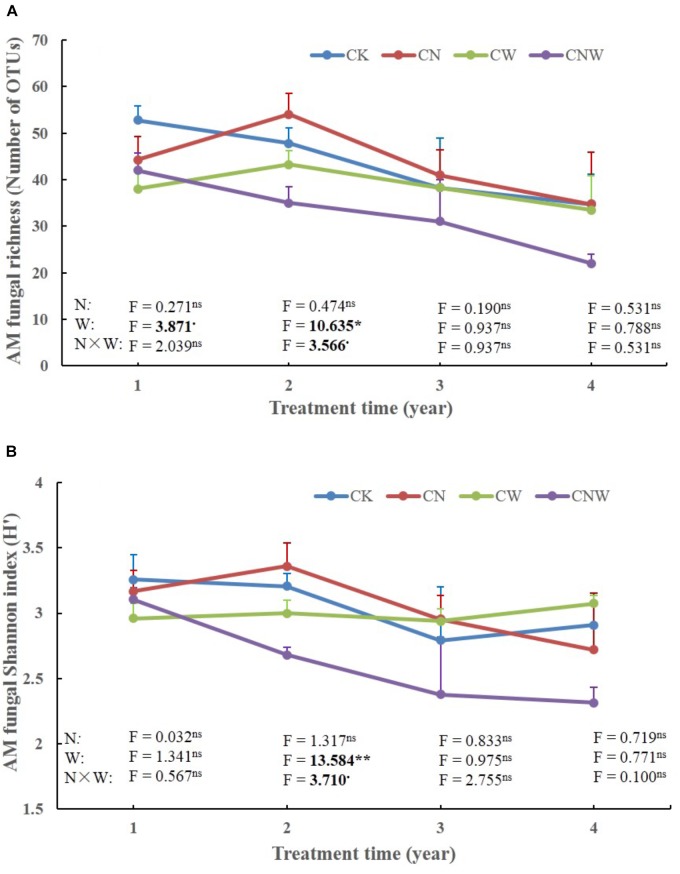
Effects of canopy additions of N and water on AM fungal richness **(A)** and Shannon diversity index (H’) **(B)** over time (4 years). CK, control; CN, canopy addition of N; CW, canopy addition of water; CNW, canopy additions of N and water. N, N addition; W, water addition; N × W, the results were the interaction. Significance of treatment effect was determined by two-way ANOVA. ^∗∗^*p* < 0.01; ^∗^*p* < 0.05; ^⋅^*p* < 0.1; ns, not significant.

**FIGURE 3:**
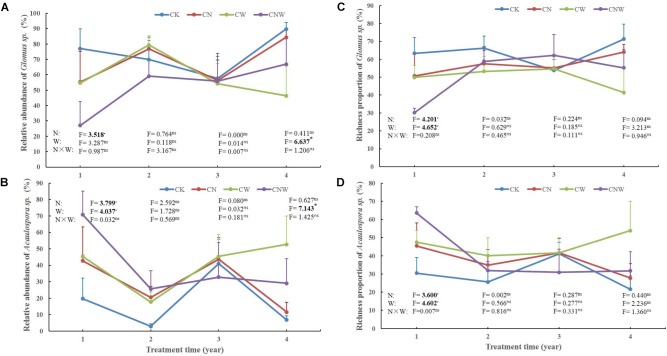
Effects of canopy additions of N and water on the abundance and richness of *Glomus sp.* **(A,C)** and *Acaulospora* *sp.* **(B,D)** over time (4 years). CK, control; CN, canopy addition of N; CW, canopy addition of water; CNW, canopy additions of N and water. N, N addition; W, water addition; N × W, the interaction. Significance of treatment effect was determined by two-way ANOVA. ^∗∗^*p* < 0.01; ^∗^*p* < 0.05; ^⋅^*p* < 0.1; ns, not significant.

In the 1st year, both N and water addition significantly changed AM fungal community composition. There was also significant interactive effect of N and water addition on AM fungal community composition (Tables 2, 3). However, no significant treatment effects were detected on AM fungal alpha-diversity (Figure 2). Moreover, N addition marginally decreased the relative abundance of *Glomus* (Figure 3A) but marginally increased *Acaulospora* relative abundance (Figure 3B). Simultaneously, water addition also marginally increased the relative abundance of *Acaulospora*, but showed no significant effect on *Glomus*. No significant interactions were found of N and water additions on the relative abundance of these two dominant genera.

**Table 2:** Effects of canopy addition of nitrogen and water on AM fungal community composition.

		1st year		2nd year		3rd year		4th year	
	d.f.	*F*	*p*	*F*	*p*	*F*	*p*	*F*	*p*
N	1	2.125	**0.037**	0.519	0.900	0.911	0.499	0.661	0.763
W	1	2.106	**0.037**	1.777	0.082	0.959	0.456	2.188	**0.029**
N × W	1	2.015	**0.047**	1.031	0.398	0.996	0.433	0.835	0.577

*F-values and p-values are presented, significant effects (*p* < 0.05) are highlighted in bold as determined by PERMANOVA. N, nitrogen addition; W, water addition; N × W, the interaction*.

**Table 3:** The dissimilarity analysis of AM fungal community composition among different treatments.

	MRPP		ADONIS		ANOSIM	
	Statistic	*p*-value	Statistic	*p*-value	Statistic	*p*-value
AM fungal community of the 1st year	0.647	**0.008**	2.014	**0.005**	0.304	**0.004**
AM fungal community of the 2nd year	0.753	0.347	1.041	0.396	-0.003	0.465
AM fungal community of the 3rd year	0.756	0.645	0.824	0.690	-0.066	0.692
AM fungal community of the 4th year	0.773	0.281	1.154	0.277	0.046	0.303

*Statistics and *p*-values are presented, significant effects (*p* < 0.05) are highlighted in bold*.

In the 2nd year, water addition significantly decreased AM fungal richness and Shannon index (Figure 2), but did not change AM fungal community composition (Tables 2, 3). By contrast, N addition showed no significant effect on AM fungal alpha-diversity or community composition (Figure 2 and Tables 2, 3). Also no significant interactions between N and water addition were observed on AM fungal alpha-diversity or community composition (Figure 2 and Table 2). In addition, both N and water additions showed marginally positive effects to the richness of *Acaulospora*, in contrast, the marginally negative effects were found on *Glomus* richness (Figures 3C,D).

In the next 2 years, both N and water additions did not show significant effects on AM fungal alpha-diversity or community composition, with an exception that water addition significantly changed AM fungal community composition, accompanied by significant raise in *Acaulospora* abundance and significant decline in that of *Glomus* (Figures 2, 3A,B and Table 2).

### Correlation Between AM Fungal Richness, Shannon Index, Community Composition Dissimilarity and Soil Properties

Only soil NH_4_^+^-N and N/P were marginally positively correlated with AM fungal richness (Table 4), and significantly positively correlated with AM fungal Shannon index (Table 4). No other significant correlations were observed between AM fungal biodiversity and soil properties.

**Table 4:** Pearson correlation analysis between AM fungal richness, Shannon index (H’) and soil properties in the 2nd year.

		Richness		Shannon index (H’)	
	d.f.	r	*p*	r	*p*
AP (mg/kg)	12	-0.272	0.826	-0.434	0.939
pH	12	0.023	0.469	0.066	0.412
SOC (g/kg)	13	-0.041	0.558	-0.093	0.629
TN (g/kg)	13	0.279	0.157	0.150	0.297
C/N	13	-0.309	0.869	-0.267	0.832
Moisture (%)	13	0.044	0.438	0.079	0.390
NH_4_^+^-N (mg/kg)	12	0.443	0.056	0.499	**0.035**
NO_3_^-^-N (mg/kg)	13	0.012	0.483	0.141	0.308
AN (mg/kg)	12	0.360	0.103	0.428	0.063
N/P	11	0.402	0.087	0.519	**0.035**

*Correlation coefficients and *p*-values are presented, significant effects (*p* < 0.05) are highlighted in bold*.

Mantel and partial Mantel test indicated that only soil pH showed marginally positive correlation with the dissimilarity among AM fungal community composition among different treatments (Table 5).

**Table 5:** Mantel and partial Mantel test of AM fungal community composition with the matrices of soil properties in the 1st year.

	Mantel test		Partial Mantel test	
	*r*	*p*	*r*	*p*
AP (mg/kg)	-0.043	0.600	-0.074	0.731
pH	0.251	0.077	0.257	0.076
SOC (g/kg)	-0.05	0.601	-0.054	0.607
TN (g/kg)	0.002	0.474	0.000	0.480
C/N	-0.209	0.969	-0.218	0.976
Moisture (%)	0.082	0.235	0.081	0.251
NH_4_^+^-N (mg/kg)	-0.055	0.616	-0.066	0.663
NO_3_^-^-N (mg/kg)	0.071	0.255	0.072	0.242
AN (mg/kg)	0.005	0.466	-0.004	0.493
N/P	0.019	0.397	0.007	0.438

*Correlation coefficients and *p*-values are presented*.

## Discussion

This study investigated the effects of canopy additions of N and water on AM fungal diversity and community composition for consecutive 4 years and the results indicated that canopy N addition significantly changed AM fungal community composition, however, this effect was time-dependent, only occurred in the 1st year. While, the effect of water addition overwhelmed that of N addition, which not only changed the community composition, but also decreased the alpha-diversity of AM fungi, and these consequences were also time-dependent and only occurred in the earlier stages (1st/2nd year). In addition, the increment of N or water tended to decrease the abundance and richness of the most dominant genus *Glomus* and favored other AM fungi. The effects of N/water addition on AM fungal community composition were potentially mediated by soil properties, such as pH, NH_4_^+^-N and N/P.

### Effects of Canopy N and Water Addition on Soil Properties

Nitrogen deposition usually leads to soil acidification (Lu et al., 2014; Tian and Niu, 2015; Chen et al., 2017), our study was not an exception (N addition significantly decreased soil pH in the 1st year), although we differently practiced canopy N addition. Possible reasons for soil acidification resulting from N deposition include: (1) NH_4_^+^ ions are absorbed by plant roots, while H^+^ will be released into soil, causing soil acidification (Smith and Read, 2008); (2) NH_4_^+^ ions are converted into nitrites and further converted into nitrates, producing H^+^ leading to soil acidification (Azevedo et al., 2013); (3) NH_4_^+^ ions displacing base cations (Ca^2+^, Mg^2+^, K^+^, Na^+^) and the increasing loss of metal cations could reduce soil buffering capacity against acidification (Tian and Niu, 2015; Lucas et al., 2016). Following soil acidification, soil microbial community composition and activity could be changed (Wei et al., 2013). Soil acidification can also result in the loss of plant species across multiple ecosystems (Azevedo et al., 2013) and the suppression of plant growth and carbon (C) sequestration (Schulte-Uebbing and de Vries, 2018).

In the 2nd year, water addition significantly decreased soil NH_4_^+^-N and AN, which may attributed to significant leaching (Martínez-García et al., 2017) and runoff. The loss of N can cause negative impacts to the environment and human, such as eutrophication of water body and decline of crop productivity, which will likely be aggravated by intensive heavy precipitation events (Martínez-García et al., 2017). On the other hand, N addition significantly increased soil NH_4_^+^-N, consistent with many previous studies (Chen et al., 2014, 2017; Zhang et al., 2014). The increase of soil NH_4_^+^-N can increase the productivity of N-limited ecosystems such as grassland and forest in temperate zone (Aber et al., 1998; Bai et al., 2010); However, excessive N supply can also lead to the accumulation of reactive nitrogen in soil to a toxic level for plant (Wei et al., 2013) and other soil organisms, such as nematodes and fungi (Eno et al., 1955). In the study of Wei et al. (2013), NH_4_^+^ concentration showed negative relationships with plant composition; in Eno et al. (1955), the fungi and nematode numbers were decreased under all N addition levels. Compared to control, only 0.6% of the nematodes and 4.9% of the fungi survived under N addition level of 608 mg kg^-1^.

### Effects of Canopy N and Water Addition on AM Fungal Alpha-Diversity

In our study, N addition did not significantly decrease AM fungal richness and Shannon index, which failed to support our first hypothesis, also inconsistent with previous studies in forest (Camenzind et al., 2014), agriculture (Liu et al., 2014) and alpine meadow ecosystems (Zheng et al., 2014). In the study of Camenzind et al. (2014) and Liu et al. (2014), N addition significantly decreased AM fungal richness, while in the study of Zheng et al. (2014), N addition had significant positive effect on AM fungal alpha-diversity. How AM fungi respond to N addition is probably influenced by local environmental conditions, plant communities, intensity and frequency of N addition, experimental duration and other unknown factors (Porras-Alfaro et al., 2007; Wang et al., 2018). In this study, the ecosystem type is forest, which has higher species diversity and stability (strong resistance) than meadow, and agriculture ecosystems. More importantly, the mode of N application in our study was canopy spraying, different from Camenzind et al. (2014), in which N was directly added to the soil. As known, many canopy processes could substantially affect the consequences of N addition, however, the extent of the impact has not yet been clarified. In addition, although the total amount of N applied in Camenzind et al. (2014) was the same as this study, but the frequency of N application was different (7 times a year in this study, versus twice a year in Camenzind’s). Low frequency with high rate could very likely over-estimate the effect of N deposition, as Zhang et al. (2014) confirmed the overestimation of plant species loss of N addition at high rates and low frequency in a temperate steppe.

In the present study, water addition significantly decreased AM fungal richness and Shannon diversity index in the 2nd year, in support of our first hypothesis, also consistent with Gao et al. (2016) and Chen et al. (2017) in steppe ecosystem. By Pearson correlation analysis, we found that NH_4_^+^-N and N/P were marginally positively correlated with AM fungal richness and significantly positively correlated with AM fungal Shannon diversity index. Meanwhile, NH_4_^+^-N and N/P were indeed significantly decreased after water addition, possibly due to run-off and leaching of N from soil (Martínez-García et al., 2017). The decrease of NH_4_^+^-N may have intensified the competition among species leading to the loss of AM fungal niche, while lost niche could finally lead to decrease of AM fungal diversity (Dickie, 2007; Gao and Guo, 2013). Moreover, water addition could affect soil nutrient balance including N/P ratio, which can largely affect AM fungal community composition (Verbruggen et al., 2015).

### Effects of Canopy N and Water Addition on AM Fungal Composition

In the 1st year, N addition significantly changed AM fungal community composition, in support of our first hypothesis, and consistent with van Diepen et al. (2011) and Camenzind et al. (2014), although the mode of N addition were different. The underlying mechanisms for the N effects on AM fungal community composition could be: (1) N addition increased the availability of soil N and reduced the cost in uptake of N by plant, so the plant dependence on mycorrhizal fungi decreased, and the amount of C allocated to mycorrhiza also decreased, which finally strengthened the competition among AM fungal species, led to changes in AM fungal community composition (Huang et al., 2014). (2) N addition led to soil acidification, which can directly affect spore germination and mycelial development (Rousk et al., 2010). More importantly, different AM fungi prefer different optimum pH, so changes in soil pH may lead to changes in community composition of AM fungi (An et al., 2008). Soil acidification caused by N addition may have stronger direct influence on soil microbial community composition than indirectly through the changes in plant community (Wei et al., 2013). At the same time, AM fungal community composition was also significantly altered by water addition, supported our first hypothesis, and in agreement with Gao et al. (2016) and Chen et al. (2017) although their studies were carried out in water-limited ecosystems. Precipitation increment may directly change soil water status and affect the physiological activity of AM fungi. Furthermore, increased precipitation can indirectly affect AM fungi via influencing the soil characteristics and plant communities. For example, in the study of Chen et al. (2017), changes in soil pH and plant species richness could shift AM fungal community composition. Gao et al. (2016) found that increased precipitation could alter fungal community composition through influencing soil moisture, NO_3_^-^-N and root turnover.

The significant interactions between N and water addition on AM fungal community composition confirmed our second hypothesis, but inconsistent with Li et al. (2015) who observed no significant interactive effect of N and water increment in a semiarid grassland ecosystem after 8 years of experimental treatment. Chen et al. (2017) found that although there was no significant interactive effect of N and precipitation increment on AM fungal diversity, but significant interactive effect was observed on the relative abundance of some AM fungal OTUs. One possible explanation was experimental duration, as in our study the interaction was only observed after 1 year of treatment.

Moreover, we also confirmed that the effect of N and water addition on AM fungal community was time-dependent, in support of our third hypothesis. The results of Yang et al. (2016) demonstrated that under field conditions, AM fungal richness increased and community composition shifted after 15 days waterlogging. However, the time resolution in our study is year-based, therefore, more sampling at finer time scales is expected to test how quickly AM fungi respond to environmental changes. In addition, in a Mediterranean grassland, increased precipitation during rainy seasons significantly altered plant community and soil fungal community structure (Suttle et al., 2007; Hawkes et al., 2011), but in the dry season, fungal community did not respond to water addition in a different Mediterranean grassland (Barnard et al., 2013). Koyama et al. (2018) suggested that besides water amounts, timing of water manipulations can also be an important influencing factor, however, the present study did not involve the timing of water addition, which could be addressed in future research. In the meta-analysis by Wang et al. (2018), they found that N addition didn’t change fungal richness significantly when the experimental duration was within 5 years or longer than 10 years, but had significant influence when the treatment duration was 5 ∼10 years. This study only lasted for 4 years, next we will continue to sample and study the long-term ecological effects of increased N deposition and precipitation.

Changes in the composition and structure of plant community may affect the amount and quality of C input to belowground thus affecting soil microbial biomass, activity, and community structure (Meier and Bowman, 2008; Treseder, 2008; Liu et al., 2016). For instance, in the meta-analysis of Liu et al. (2016), plant lignin, plant protein and soil lignin were significantly increased by 7.13, 25.94, 7.30%, respectively following N addition. On the one hand, the increase of litter quality could promote microbial growth and biomass accumulation; on the other hand, the increase of recalcitrant C compounds (e.g., lignin) could result in the decrease of C availability to soil microbes, inhibiting microbial growth and activity (Treseder, 2008). Moreover, community composition of some specific microbial groups can change under N additions, for example, the diversity of ectomycorrhizal fungi and the richness of fungal decomposers decreased after N fertilization or deposition (Treseder, 2008). It should be further noted that, as symbiotic fungi, AM fungi have host preference (Sanders, 2003; Croll et al., 2008), and their community composition and structure are closely linked to plant community characteristics (Öpik et al., 2010; Kivlin et al., 2011; Xu et al., 2016). At the regional scale, a significant relationship between AM fungal community composition and plant was observed by Xu et al. (2016). The results of Li et al. (2015) indicated that the AM fungal abundance and OTU richness were significantly correlated with the 7-year averaged ANPP and aboveground biomass of plant functional groups after 8-years N and water additions. In the study of Chen et al. (2017), significant correlation between plant species richness and AM fungal taxonomic composition was also recorded. Therefore, further research incorporating plant community data is still needed.

## Conclusion

Increased N deposition and precipitation have significant interactive effect on AM fungal diversity and community composition, while precipitation increment have stronger effect on AM fungal community structure than increased N deposition in the forest ecosystem. The effect of N deposition and precipitation on AM fungal community composition was time-dependent, mediated by soil factors, and possibly related to the sensitivity and resilience of forest ecosystem to global changes. In the future, we will consider finer and broader time scales and take into account the plant data to achieve comprehensive understanding of AM fungal ecology in the forest ecosystem.

## Author Contributions

AZ conducted the experiments, analyzed the data, and drafted the manuscript. LL conceived the study, received financial support, and conducted parts of the experiments. BC, TX, and WX performed the data analysis. LS provided some basic data. WZ was responsible for the operation of this experimental platform. SF and HF designed and established this experimental platform. BC revised the manuscript.

## Conflict of Interest Statement

The authors declare that the research was conducted in the absence of any commercial or financial relationships that could be construed as a potential conflict of interest.
