# Supplementary material for: Influences of Canopy Nitrogen and Water Addition on AM Fungal Biodiversity and Community Composition in a Mixed Deciduous Forest of China
Source: Front Plant Sci. 2018 Dec 11;9:1842. doi: 10.3389/fpls.2018.01842 (PMC6297361; doi:10.3389/fpls.2018.01842)
Supplement: Supplementary file 1 [file Table_1.docx]

**Influences of canopy nitrogen and water additions on AM fungal biodiversity and community composition in a mixed deciduous forest of China**

Aihua Zhao, Lei Liu, Tianle Xu, Leilei Shi, Wei Xie, Wei Zhang, Shenglei Fu, Haiyan Feng, Baodong Chen ^*^

* Author for correspondence: Baodong Chen

E-mail: bdchen@rcees.ac.cn; Tel: +86 10 62849068

Address: Research Center for Eco-Environmental Sciences, 18 Shuangqinglu, Haidian District, Beijing 100085, China

***Supplementary Information***

**Table S1~ Table S2**

**Figure S1**

**Table S1** List of AM fungal molecular taxa

| OTUs | MaarjAM  (best hit) | Family | Genus | Max identity (%) | |
| --- | --- | --- | --- | --- | --- |
| OTU1 | VTX00227 | Acaulosporaceae | *Acaulospora* | | 99.5 |
| OTU2 | VTX00270 | Glomeraceae | *Glomus* | | 99.5 |
| OTU3 | VTX00166 | Glomeraceae | *Glomus* | | 100 |
| OTU4 | VTX00024 | Acaulosporaceae | *Acaulospora* | | 100 |
| OTU5 | VTX00219 | Glomeraceae | *Glomus* | | 99.5 |
| OTU6 | VTX00227 | Acaulosporaceae | *Acaulospora* | | 98.2 |
| OTU7 | VTX00166 | Glomeraceae | *Glomus* | | 99.5 |
| OTU8 | VTX00219 | Glomeraceae | *Glomus* | | 99.1 |
| OTU9 | VTX00080 | Glomeraceae | *Glomus* | | 99.5 |
| OTU10 | VTX00270 | Glomeraceae | *Glomus* | | 99.5 |
| OTU11 | VTX00227 | Acaulosporaceae | *Acaulospora* | | 99.1 |
| OTU12 | VTX00219 | Glomeraceae | *Glomus* | | 100 |
| OTU13 | VTX00080 | Glomeraceae | *Glomus* | | 100 |
| OTU14 | VTX00080 | Glomeraceae | *Glomus* | | 98.6 |
| OTU15 | VTX00193 | Claroideoglomeraceae | *Claroideoglomus* | | 100 |
| OTU16 | VTX00024 | Acaulosporaceae | *Acaulospora* | | 100 |
| OTU17 | VTX00270 | Glomeraceae | *Glomus* | | 99.1 |
| OTU18 | VTX00268 | Glomeraceae | *Glomus* | | 100 |
| OTU19 | VTX00219 | Glomeraceae | *Glomus* | | 99.5 |
| OTU20 | VTX00096 | Glomeraceae | *Glomus* | | 100 |
| OTU21 | VTX00069 | Glomeraceae | *Glomus* | | 100 |
| OTU22 | VTX00113 | Glomeraceae | *Glomus* | | 100 |
| OTU23 | VTX00024 | Acaulosporaceae | *Acaulospora* | | 98.6 |
| OTU24 | VTX00412 | Glomeraceae | *Glomus* | | 97.7 |
| OTU25 | VTX00219 | Glomeraceae | *Glomus* | | 100 |
| OTU26 | VTX00159 | Glomeraceae | *Glomus* | | 99.1 |
| OTU27 | VTX00024 | Acaulosporaceae | *Acaulospora* | | 99.5 |
| OTU28 | VTX00370 | Glomeraceae | *Glomus* | | 100 |
| OTU29 | VTX00219 | Glomeraceae | *Glomus* | | 99.1 |
| OTU30 | VTX00166 | Glomeraceae | *Glomus* | | 99.5 |
| OTU31 | VTX00270 | Glomeraceae | *Glomus* | | 99.1 |
| OTU32 | VTX00278 | Claroideoglomeraceae | *Claroideoglomus* | | 99.6 |
| OTU33 | VTX00310 | Glomeraceae | *Glomus* | | 98.1 |
| OTU34 | VTX00024 | Acaulosporaceae | *Acaulospora* | | 99.1 |
| OTU35 | VTX00113 | Glomeraceae | *Glomus* | | 100 |
| OTU36 | VTX00219 | Glomeraceae | *Glomus* | | 99.1 |
| OTU37 | VTX00080 | Glomeraceae | *Glomus* | | 99.5 |
| OTU38 | VTX00159 | Glomeraceae | *Glomus* | | 99.1 |
| OTU39 | VTX00049 | Gigasporaceae | *Scutellospora* | | 100 |
| OTU40 | VTX00156 | Glomeraceae | *Glomus* | | 100 |
| OTU41 | VTX00024 | Acaulosporaceae | *Acaulospora* | | 99.5 |
| OTU42 | VTX00166 | Glomeraceae | *Glomus* | | 99.1 |
| OTU43 | VTX00096 | Glomeraceae | *Glomus* | | 100 |
| OTU44 | VTX00039 | Gigasporaceae | *Gigaspora* | | 99.5 |
| OTU45 | VTX00024 | Acaulosporaceae | *Acaulospora* | | 99.1 |
| OTU46 | VTX00310 | Glomeraceae | *Glomus* | | 98.6 |
| OTU47 | VTX00130 | Glomeraceae | *Glomus* | | 100 |
| OTU48 | VTX00092 | Glomeraceae | *Glomus* | | 99.5 |
| OTU49 | VTX00166 | Glomeraceae | *Glomus* | | 99.5 |
| OTU50 | VTX00227 | Acaulosporaceae | *Acaulospora* | | 99.1 |
| OTU51 | VTX00191 | Glomeraceae | *Glomus* | | 99.1 |
| OTU52 | VTX00057 | Claroideoglomeraceae | *Claroideoglomus* | | 99.6 |
| OTU53 | VTX00024 | Acaulosporaceae | *Acaulospora* | | 99.5 |
| OTU54 | VTX00072 | Glomeraceae | *Glomus* | | 98.6 |
| OTU55 | VTX00227 | Acaulosporaceae | *Acaulospora* | | 99.1 |
| OTU56 | VTX00310 | Glomeraceae | *Glomus* | | 98.1 |
| OTU57 | VTX00024 | Acaulosporaceae | *Acaulospora* | | 99.1 |
| OTU58 | VTX00166 | Glomeraceae | *Glomus* | | 99.1 |
| OTU59 | VTX00125 | Glomeraceae | *Glomus* | | 100 |
| OTU60 | VTX00156 | Glomeraceae | *Glomus* | | 99.5 |
| OTU61 | VTX00219 | Glomeraceae | *Glomus* | | 98.6 |
| OTU62 | VTX00063 | Glomeraceae | *Glomus* | | 97.7 |
| OTU63 | VTX00159 | Glomeraceae | *Glomus* | | 99.5 |
| OTU64 | VTX00222 | Glomeraceae | *Glomus* | | 100 |
| OTU65 | VTX00039 | Gigasporaceae | *Gigaspora* | | 100 |
| OTU66 | VTX00227 | Acaulosporaceae | *Acaulospora* | | 99.1 |
| OTU67 | VTX00227 | Acaulosporaceae | *Acaulospora* | | 99.5 |
| OTU68 | VTX00069 | Glomeraceae | *Glomus* | | 98.1 |
| OTU69 | VTX00214 | Glomeraceae | *Glomus* | | 100 |
| OTU70 | VTX00126 | Glomeraceae | *Glomus* | | 99.5 |
| OTU71 | VTX00270 | Glomeraceae | *Glomus* | | 99.1 |
| OTU72 | VTX00222 | Glomeraceae | *Glomus* | | 99.5 |
| OTU73 | VTX00113 | Glomeraceae | *Glomus* | | 99.1 |
| OTU74 | VTX00281 | Paraglomeraceae | *Paraglomus* | | 100 |
| OTU75 | VTX00049 | Gigasporaceae | *Scutellospora* | | 99.5 |
| OTU76 | VTX00223 | Glomeraceae | *Glomus* | | 99.5 |
| OTU77 | VTX00219 | Glomeraceae | *Glomus* | | 99.1 |
| OTU78 | VTX00227 | Acaulosporaceae | *Acaulospora* | | 99.5 |
| OTU79 | VTX00080 | Glomeraceae | *Glomus* | | 99.1 |
| OTU80 | VTX00242 | Ambisporaceae | *Ambispora* | | 99.1 |
| OTU81 | VTX00222 | Glomeraceae | *Glomus* | | 100 |
| OTU82 | VTX00039 | Gigasporaceae | *Gigaspora* | | 99.1 |
| OTU83 | VTX00212 | Glomeraceae | *Glomus* | | 99.1 |
| OTU84 | VTX00270 | Glomeraceae | *Glomus* | | 98.1 |
| OTU85 | VTX00222 | Glomeraceae | *Glomus* | | 99.5 |
| OTU86 | VTX00024 | Acaulosporaceae | *Acaulospora* | | 98.6 |
| OTU87 | VTX00239 | Paraglomeraceae | *Paraglomus* | | 99.5 |
| OTU88 | VTX00115 | Glomeraceae | *Glomus* | | 98.1 |
| OTU89 | VTX00092 | Glomeraceae | *Glomus* | | 99.1 |
| OTU90 | VTX00024 | Acaulosporaceae | *Acaulospora* | | 99.5 |
| OTU91 | VTX00159 | Glomeraceae | *Glomus* | | 100 |
| OTU92 | VTX00024 | Acaulosporaceae | *Acaulospora* | | 100 |
| OTU93 | VTX00227 | Acaulosporaceae | *Acaulospora* | | 97.7 |
| OTU94 | VTX00227 | Acaulosporaceae | *Acaulospora* | | 98.2 |
| OTU95 | VTX00076 | Glomeraceae | *Glomus* | | 99.5 |
| OTU96 | VTX00370 | Glomeraceae | *Glomus* | | 99.1 |
| OTU97 | VTX00227 | Acaulosporaceae | *Acaulospora* | | 99.1 |
| OTU98 | VTX00166 | Glomeraceae | *Glomus* | | 98.1 |
| OTU99 | VTX00227 | Acaulosporaceae | *Acaulospora* | | 99.1 |
| OTU100 | VTX00080 | Glomeraceae | *Glomus* | | 99.1 |
| OTU101 | VTX00166 | Glomeraceae | *Glomus* | | 99.5 |
| OTU102 | VTX00096 | Glomeraceae | *Glomus* | | 99.5 |
| OTU103 | VTX00113 | Glomeraceae | *Glomus* | | 100 |
| OTU104 | VTX00063 | Glomeraceae | *Glomus* | | 100 |
| OTU105 | VTX00214 | Glomeraceae | *Glomus* | | 99.5 |
| OTU106 | VTX00105 | Glomeraceae | *Glomus* | | 99.5 |
| OTU107 | VTX00024 | Acaulosporaceae | *Acaulospora* | | 99.1 |
| OTU108 | VTX00222 | Glomeraceae | *Glomus* | | 100 |
| OTU109 | VTX00278 | Claroideoglomeraceae | *Claroideoglomus* | | 100 |
| OTU110 | VTX00227 | Acaulosporaceae | *Acaulospora* | | 98.2 |
| OTU111 | VTX00004 | Archaeosporaceae | *Archaeospora* | | 100 |
| OTU112 | VTX00156 | Glomeraceae | *Glomus* | | 99.5 |
| OTU113 | VTX00080 | Glomeraceae | *Glomus* | | 99.1 |
| OTU114 | VTX00156 | Glomeraceae | *Glomus* | | 99.1 |
| OTU115 | VTX00096 | Glomeraceae | *Glomus* | | 99.1 |
| OTU116 | VTX00024 | Acaulosporaceae | *Acaulospora* | | 99.5 |
| OTU117 | VTX00353 | Diversisporaceae | *Diversispora* | | 98.2 |
| OTU118 | VTX00270 | Glomeraceae | *Glomus* | | 99.5 |
| OTU119 | VTX00166 | Glomeraceae | *Glomus* | | 98.6 |
| OTU120 | VTX00227 | Acaulosporaceae | *Acaulospora* | | 97.7 |
| OTU121 | VTX00219 | Glomeraceae | *Glomus* | | 99.5 |
| OTU122 | VTX00268 | Glomeraceae | *Glomus* | | 99.5 |
| OTU123 | VTX00004 | Archaeosporaceae | *Archaeospora* | | 98.6 |
| OTU124 | VTX00113 | Glomeraceae | *Glomus* | | 98.6 |
| OTU125 | VTX00004 | Archaeosporaceae | *Archaeospora* | | 100 |
| OTU126 | VTX00310 | Glomeraceae | *Glomus* | | 98.1 |
| OTU127 | VTX00122 | Glomeraceae | *Glomus* | | 99.5 |
| OTU128 | VTX00340 | Claroideoglomeraceae | *Claroideoglomus* | | 97.3 |
| OTU129 | VTX00166 | Glomeraceae | *Glomus* | | 98.6 |
| OTU130 | VTX00092 | Glomeraceae | *Glomus* | | 99.1 |
| OTU131 | VTX00219 | Glomeraceae | *Glomus* | | 100 |
| OTU132 | VTX00270 | Glomeraceae | *Glomus* | | 99.5 |
| OTU133 | VTX00270 | Glomeraceae | *Glomus* | | 98.6 |
| OTU134 | VTX00124 | Glomeraceae | *Glomus* | | 99.1 |
| OTU135 | VTX00340 | Claroideoglomeraceae | *Claroideoglomus* | | 98.2 |
| OTU136 | VTX00227 | Acaulosporaceae | *Acaulospora* | | 99.5 |
| OTU137 | VTX00024 | Acaulosporaceae | *Acaulospora* | | 99.5 |
| OTU138 | VTX00024 | Acaulosporaceae | *Acaulospora* | | 99.5 |
| OTU139 | VTX00080 | Glomeraceae | *Glomus* | | 98.6 |
| OTU140 | VTX00199 | Glomeraceae | *Glomus* | | 100 |
| OTU141 | VTX00039 | Gigasporaceae | *Gigaspora* | | 99.1 |
| OTU142 | VTX00227 | Acaulosporaceae | *Acaulospora* | | 99.1 |
| OTU143 | VTX00024 | Acaulosporaceae | *Acaulospora* | | 99.1 |
| OTU144 | VTX00113 | Glomeraceae | *Glomus* | | 99.5 |
| OTU145 | VTX00077 | Glomeraceae | *Glomus* | | 98.6 |
| OTU146 | VTX00383 | Glomeraceae | *Glomus* | | 99.5 |

**Table S2** Soil properties

|  | 1^st^ year | | | | | | | | | | | |
| --- | --- | --- | --- | --- | --- | --- | --- | --- | --- | --- | --- | --- |
|  | CK | | | CN | | | CW | | | CNW | | |
| AP (mg/kg) | 44.11 | ± | 6.84a | 34.90 | ± | 10.85a | 45.34 | ± | 3.22a | 39.81 | ± | 8.22a |
| pH | 4.57 | ± | 0.12a | 4.29 | ± | 0.09b | 4.47 | ± | 0.03ab | 4.32 | ± | 0.04ab |
| SOC (g/kg) | 40.85 | ± | 1.99a | 50.27 | ± | 4.44a | 46.30 | ± | 2.31a | 49.77 | ± | 3.42a |
| TN ((g/kg) | 2.47 | ± | 0.32a | 2.75 | ± | 0.07a | 2.59 | ± | 0.18a | 2.29 | ± | 0.16a |
| C/N | 18.95 | ± | 1.54a | 18.29 | ± | 1.51a | 17.97 | ± | 0.53a | 21.89 | ± | 1.61a |
| Moisture (%) | 13.96 | ± | 1.46a | 13.79 | ± | 1.64a | 13.35 | ± | 0.80a | 15.04 | ± | 1.06a |
| NH_4_^+^-N (mg/kg) | 10.04 | ± | 0.94a | 11.39 | ± | 1.58a | 11.14 | ± | 0.99a | 13.01 | ± | 2.83a |
| NO_3_^-^-N (mg/kg) | 14.72 | ± | 2.79a | 14.98 | ± | 2.24a | 11.97 | ± | 1.82a | 16.63 | ± | 1.61a |
| AN (mg/kg) | 24.76 | ± | 2.23a | 26.37 | ± | 2.40a | 23.11 | ± | 2.22a | 29.63 | ± | 3.05a |
| N/P | 0.63 | ± | 0.16a | 1.11 | ± | 0.46a | 0.52 | ± | 0.07a | 0.58 | ± | 0.06a |
|  | 2^nd^ year | | | | | | | | | | | |
|  | CK | | | CN | | | CW | | | CNW | | |
| AP (mg/kg) | 22.44 | ± | 0.63a | 27.57 | ± | 2.42a | 30.6 | ± | 2.06a | 30.88 | ± | 3.36a |
| pH | 4.35 | ± | 0.15a | 4.25 | ± | 0.10a | 4.29 | ± | 0.08a | 4.22 | ± | 0.03a |
| SOC (g/kg) | 60.59 | ± | 5.32a | 64.41 | ± | 11.14a | 64.82 | ± | 8.08a | 66.17 | ± | 2.42a |
| TN ((g/kg) | 3.46 | ± | 0.29a | 3.65 | ± | 0.26a | 3.45 | ± | 0.44a | 3.41 | ± | 0.09a |
| C/N | 17.58 | ± | 0.62a | 17.44 | ± | 2.16a | 21.1 | ± | 4.20a | 19.43 | ± | 0.87a |
| Moisture (%) | 20.89 | ± | 1.09a | 20.98 | ± | 1.32a | 20.32 | ± | 1.28a | 22.07 | ± | 1.84a |
| NH_4_^+^-N (mg/kg) | 5.18 | ± | 1.24b | 8.24 | ± | 0.66a | 3.62 | ± | 0.18b | 4.23 | ± | 0.25b |
| NO_3_^-^-N (mg/kg) | 8.03 | ± | 1.00a | 8.91 | ± | 0.96a | 10.22 | ± | 2.36a | 7.59 | ± | 1.27a |
| AN (mg/kg) | 13.21 | ± | 2.11ab | 17.14 | ± | 1.19a | 11.69 | ± | 1.47b | 11.83 | ± | 1.41b |
| N/P | 0.59 | ± | 0.15a | 0.64 | ± | 0.07a | 0.38 | ± | 0.08a | 0.41 | ± | 0.08a |
|  | 3^rd^ year | | | | | | | | | | | |
|  | CK | | | CN | | | CW | | | CNW | | |
| AP (mg/kg) | 27.40 | ± | 6.56a | 32.15 | ± | 3.12a | 33.86 | ± | 5.16a | 32.34 | ± | 8.31a |
| pH | 4.45 | ± | 0.11a | 4.43 | ± | 0.10a | 4.39 | ± | 0.09a | 4.37 | ± | 0.05a |
| SOC (g/kg) | 37.10 | ± | 5.33a | 43.78 | ± | 8.86a | 31.02 | ± | 3.01a | 33.08 | ± | 2.14a |
| TN ((g/kg) | 1.70 | ± | 0.16a | 1.93 | ± | 0.27a | 1.58 | ± | 0.13a | 1.5 | ± | 0.12a |
| C/N | 21.62 | ± | 1.46a | 22.24 | ± | 1.80a | 19.76 | ± | 1.19a | 22.17 | ± | 0.91a |
| Moisture (%) | 15.39 | ± | 0.66a | 17.57 | ± | 1.64a | 17.97 | ± | 1.55a | 17.77 | ± | 0.89a |
| NH_4_^+^-N (mg/kg) | 1.98 | ± | 0.16a | 1.99 | ± | 0.14a | 3.10 | ± | 0.54a | 2.7 | ± | 0.44a |
| NO_3_^-^-N (mg/kg) | 6.27 | ± | 2.06a | 6.53 | ± | 1.57a | 8.37 | ± | 1.34a | 10.95 | ± | 1.44a |
| AN (mg/kg) | 8.24 | ± | 2.08b | 8.52 | ± | 1.56b | 11.47 | ± | 1.33ab | 13.64 | ± | 1.16a |
| N/P | 0.31 | ± | 0.05a | 0.29 | ± | 0.07a | 0.35 | ± | 0.03a | 0.56 | ± | 0.20a |
|  | 4^th^ year | | | | | | | | | | | |
|  | CK | | | CN | | | CW | | | CNW | | |
| AP (mg/kg) | 11.54 | ± | 1.88a | 16.04 | ± | 3.28a | 23.04 | ± | 1.65a | 22.94 | ± | 9.55a |
| pH | 4.41 | ± | 0.04a | 4.28 | ± | 0.07a | 4.49 | ± | 0.05a | 4.4 | ± | 0.10a |
| SOC (g/kg) | 38.37 | ± | 5.29a | 43.01 | ± | 11.86a | 33.08 | ± | 1.82a | 28.56 | ± | 1.73a |
| TN ((g/kg) | 1.68 | ± | 0.18a | 1.75 | ± | 0.22a | 1.58 | ± | 0.08a | 1.4 | ± | 0.11a |
| C/N | 22.91 | ± | 2.53a | 23.30 | ± | 3.48a | 21.00 | ± | 0.62a | 20.48 | ± | 0.52a |
| Moisture (%) | 19.15 | ± | 0.77a | 20.14 | ± | 1.47a | 18.22 | ± | 0.65a | 19.4 | ± | 1.13a |
| NH_4_^+^-N (mg/kg) | 5.35 | ± | 0.89a | 6.06 | ± | 0.79a | 4.18 | ± | 0.28a | 5.44 | ± | 0.66a |
| NO_3_^-^-N (mg/kg) | 4.28 | ± | 0.67a | 5.19 | ± | 0.94a | 3.39 | ± | 0.74a | 3.82 | ± | 0.98a |
| AN (mg/kg) | 9.63 | ± | 1.00a | 11.25 | ± | 1.60a | 7.57 | ± | 0.72a | 9.26 | ± | 1.63a |
| N/P | 0.90 | ± | 0.13a | 0.75 | ± | 0.12a | 0.33 | ± | 0.05a | 0.75 | ± | 0.34a |

Data are presented as means ± standard error (SE). Duncan’s multiple range test was performed across treatments and different letters next to SE in the same row indicate significant differences (*p* <0.05).

CK, control; CN, canopy addition of N; CW, canopy addition of water;

CNW, canopy additions of N and water.


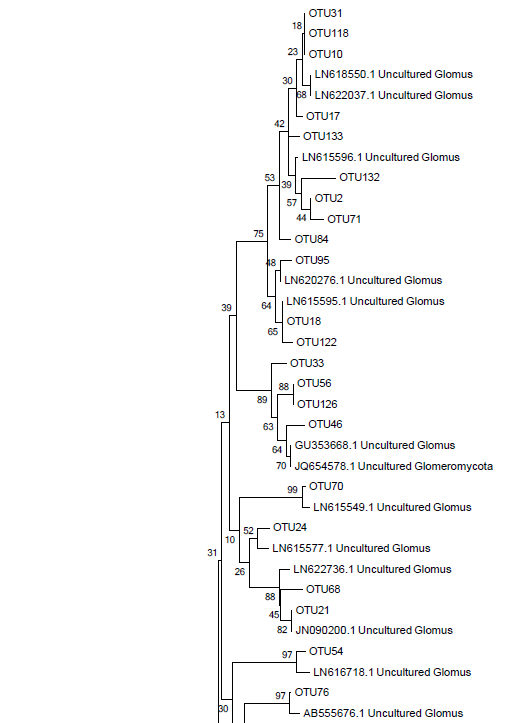

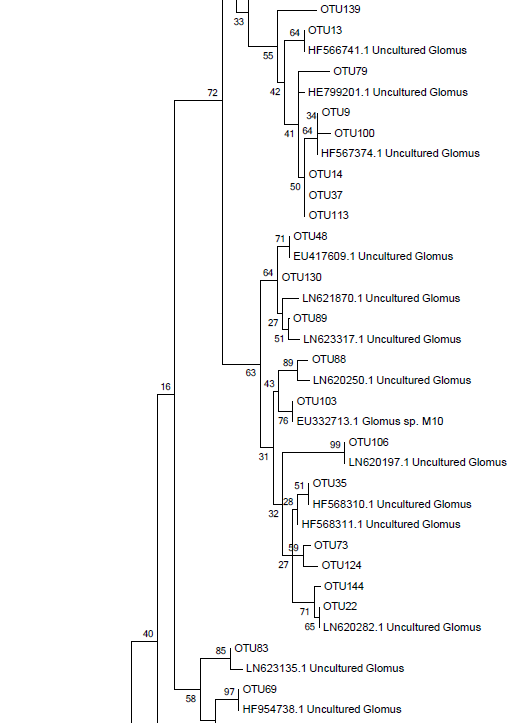

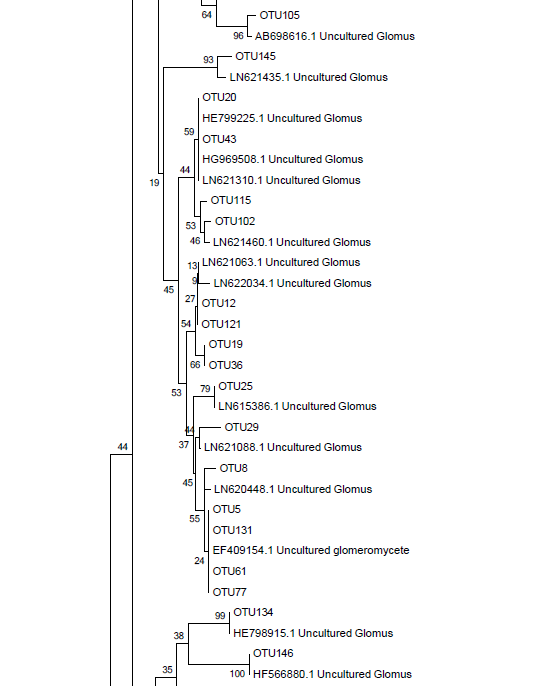

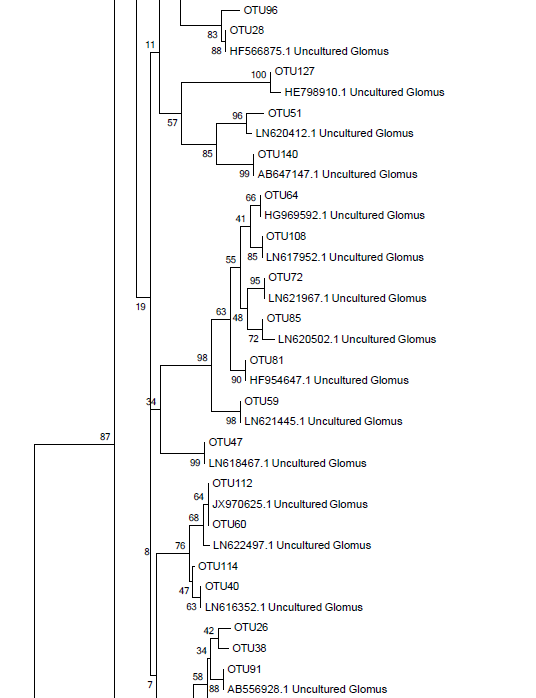

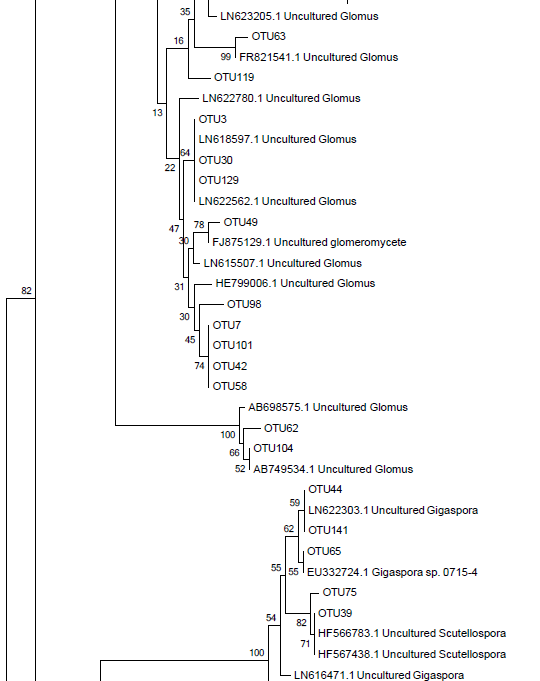

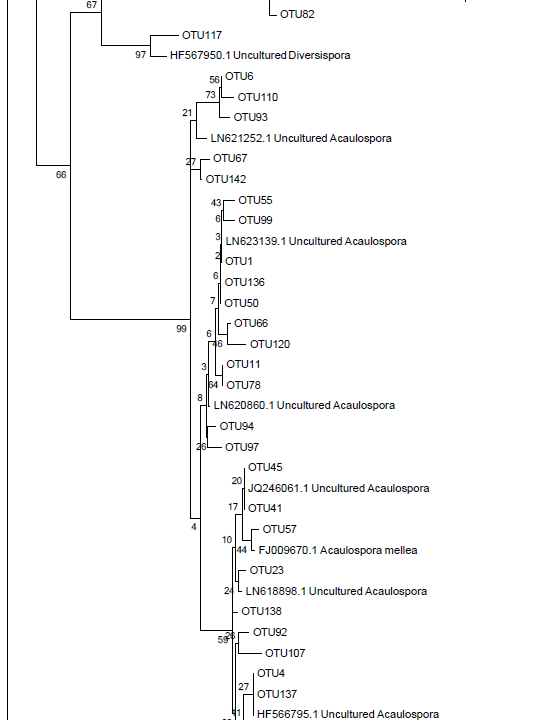

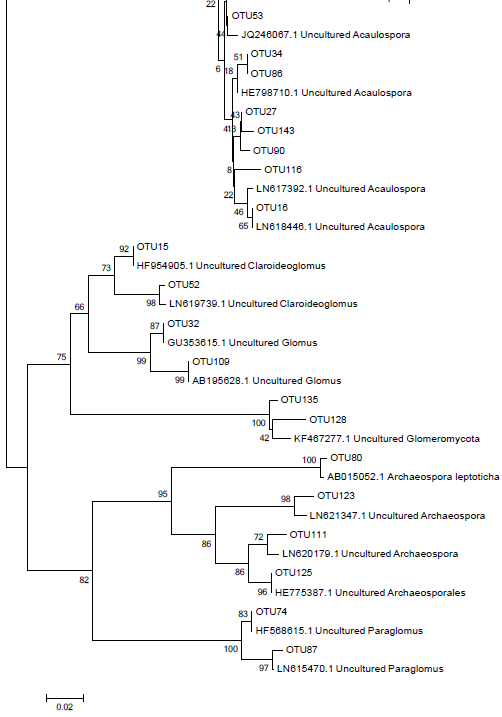


**Figure S1** Neighbor joining phylogenetic tree for the 146 AM fungal OTUs.
